# Target and biomarker exploration portal for drug discovery

**DOI:** 10.1093/bioinformatics/btaf627

**Published:** 2025-11-13

**Authors:** Bhupesh Dewangan, Debjyoti Ray, Yijie Ren, Shraddha Srivastava, Lei Jiang, Muneendra Ojha, Dong Xu, Gyan Srivastava

**Affiliations:** Indian Institute of Information Technology, Allahabad 211015, India; Indian Institute of Information Technology, Allahabad 211015, India; Department of Electrical Engineering and Computer Science and Christopher S. Bond Life Sciences Center, University of Missouri, Columbia, MO 65211, United States; Indian Institute of Information Technology, Allahabad 211015, India; Department of Electrical Engineering and Computer Science and Christopher S. Bond Life Sciences Center, University of Missouri, Columbia, MO 65211, United States; Indian Institute of Information Technology, Allahabad 211015, India; Department of Electrical Engineering and Computer Science and Christopher S. Bond Life Sciences Center, University of Missouri, Columbia, MO 65211, United States; Department of Electrical Engineering and Computer Science and Christopher S. Bond Life Sciences Center, University of Missouri, Columbia, MO 65211, United States; Bioinformatics and Data Science, Alexion Pharmaceutical, AstraZeneca, Boston, MA 2210, United States

## Abstract

**Motivation:**

The discovery of novel drug targets and precision biomarkers remains a major challenge in drug development, with traditional differential expression analysis often overlooking key regulatory proteins. Here, we present a novel, web-based bioinformatics tool, the Target and Biomarker Exploration Portal (TBEP), designed to accelerate the drug discovery process by integrating large-scale biomedical data with network analysis techniques.

**Results:**

TBEP harnesses machine-learning approaches to mine and combine multimodal datasets, including human genetics, functional genomics, and protein-protein interaction networks, to decode causal disease mechanisms and uncover novel therapeutic targets and precision biomarkers for specific phenotypes. A unique feature of the tool is its ability to process large-scale data in real-time, facilitated by an efficient cloud-based architecture. Additionally, the tool incorporates an integrated large language model (LLM), which assists researchers in exploring and interpreting complex biological relationships within the generated networks and multi-omics data using natural language (English). By offering an intuitive, interactive interface, the LLM enhances the exploration of biological insights, making it easier for scientists to derive actionable conclusions. This powerful integration of network analysis, multi-omics data, and LLM provides a robust framework for accelerating the identification of novel drug targets.

**Availability and implementation:**

The tool is publicly available at https://tbep.missouri.edu. The source code, documentation and installation instructions are available at GitHub repository: https://github.com/mizzoudbl/tbep.

## 1 Introduction

The discovery of novel drug targets and clinically actionable biomarkers remains a major challenge in the drug development pipeline. While conventional approaches, such as differential gene expression analyses, have contributed significantly to our understanding of disease biology, they often fall short in capturing the complexity of biological systems. Specifically, these methods may overlook key regulatory proteins that exert their influence at the network level rather than through measurable expression changes, leading to missed opportunities for therapeutic intervention. As the volume and diversity of biomedical data continue to grow, spanning human genetics, transcriptomics, and protein–protein interaction (PPI) networks, there is a critical need for integrative frameworks capable of translating this complexity into actionable biological insights.

Network-based computational approaches offer the ability to model biological systems as interconnected networks of genes, proteins, and pathways (Zitnik *et al.* 2024). By capturing the topological and functional architecture of disease-relevant networks, these methods can reveal critical regulatory hubs and novel targets that traditional analyses may miss. Several web-based platforms support multi-omics integration using network-based methodologies (Ewald *et al.* 2024, Sugimoto *et al.* 2024); however, significant challenges remain in managing, analyzing, and interpreting large-scale, multimodal datasets. These challenges underscore the need for advanced tools that can efficiently integrate diverse data sources while maintaining biological interpretability and translational relevance.

To address these limitations, we introduce the Target and Biomarker Exploration Portal (TBEP), a cloud-enabled, web-based bioinformatics platform designed to accelerate the identification of disease-associated targets and biomarkers. TBEP integrates multi-modal biomedical datasets, including human genetics, functional genomics, and curated PPI networks, within a scalable, AI-driven framework that supports real-time analysis and visualization of complex biological networks. A distinguishing feature of TBEP is its integration with Knowledge Bot, a specialized question-answering (QA) system built on a fine-tuned large language model (LLM). This system enables structured, citation-supported querying of biomedical concepts, facilitating the extraction of mechanistic insights and hypothesis generation directly from the platform.

By combining advanced network analysis, machine learning, and domain-specific natural language processing, TBEP offers a comprehensive and user-friendly environment for multi-omics exploration. This integration supports a broad range of applications, from early-stage target discovery to translational biomarker research, positioning TBEP as a valuable resource for both academic investigators and pharmaceutical partners seeking to advance precision medicine initiatives.

## 2 Materials and methods

**Figure 1.**
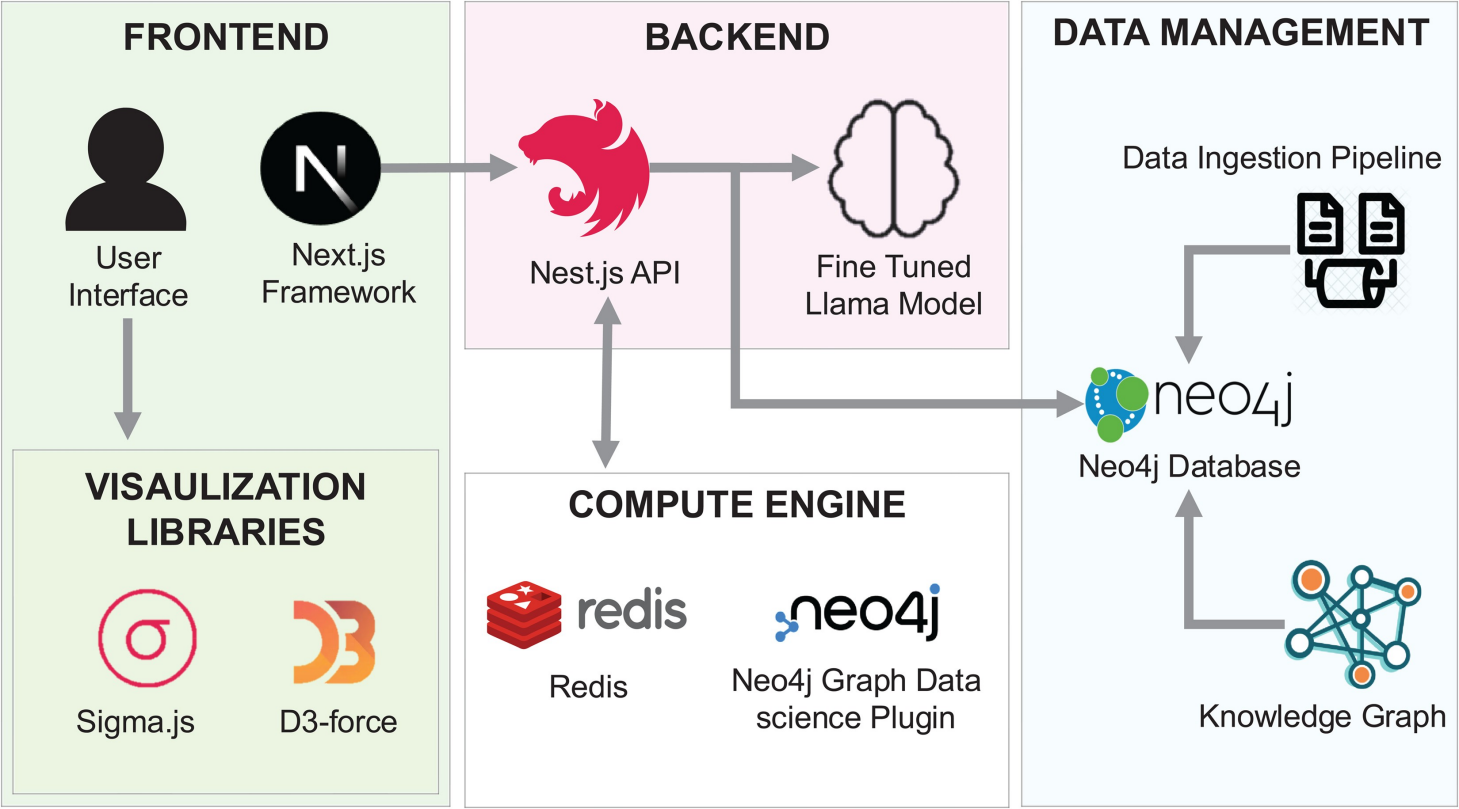
Illustration of the full-stack technologies employed in the tool’s development. The figure showcases the integration of front-end and back-end components. It also depicts the overall architecture, highlighting the connectivity and interactions among core modules for seamless system functionality.

TBEP is a web-based platform designed to facilitate the exploration and analysis of complex biological networks. By integrating diverse technologies, TBEP allows researchers to map and interpret extensive biomedical data efficiently and intuitively. The platform’s architecture consists of front-end data visualization and backend data management. Figure 1 illustrates the user interface layout, highlighting its various components. The interface integrates these elements seamlessly to facilitate efficient navigation.

### 2.1 Frontend and visualization framework

The frontend of TBEP, built with Next.js, provides a seamless environment for both client- and server-side rendering, optimizing the user experience by ensuring responsive, high-performance interactions across devices. Within this framework, Sigma.js and D3-force are key components for network visualization. Sigma.js operates on a WebGL and Canvas rendering base, which is advantageous for handling large, complex datasets with high graphical demands. Unlike SVG-based methods, WebGL provides the scalability and speed needed for real-time, interactive data manipulation.

TBEP’s network visualizations are powered by the D3-force layout algorithm, which employs realistic, physics-based forces using the Velocity Verlet numerical integrator on the network structure (https://github.com/d3/d3-force). These layouts simulate the natural organization of nodes and edges, creating clusters and connections that make complex relationships easier to interpret. Through interactive controls, users can upload data, adjust visualization settings like node color and size to reflect different omics properties and data attributes, perform algorithmic analysis, and submit queries using natural language (English), empowering them to explore biomedical networks dynamically. This level of interactivity facilitates deeper data exploration and enables users to quickly identify “hot spots” or areas of high significance within complex biological networks.

### 2.2 Backend and data management

The TBEP backend utilizes Nest.js with GraphQL as its core API framework, ensuring robust and scalable application logic. Bioinformatics data is stored in Neo4j, a graph database that represents biological entities and relationships as nodes and edges, effectively capturing the interconnected nature of biomedical data. This graph database format supports rapid, flexible querying of large datasets, ensuring efficient retrieval of data relevant to user queries and visualizations. The data ingestion pipeline processes bioinformatics data sourced from curated databases such as STRING (Szklarczyk *et al.* 2023) and Reactome (Milacic *et al.* 2024), then loads it into Neo4j, establishing a cohesive, well-organized knowledge base within TBEP.

### 2.3 Knowledge base: data sources

The tool utilizes a variety of biomedical data sources, which are integrated for comprehensive analysis. These data include:

**Reference genome data**: We used the reference genome from the HGNC database (Seal *et al.* 2023), with Ensembl ID as the primary key identifier. All subsequent data types (mentioned as follows) were aligned with the reference genome before being ingested into the knowledge base. This alignment ensures consistency and compatibility across datasets, enabling accurate integration and analysis within the system.

**PPI networks:** In the current version of the portal, protein interaction network data were curated from the STRING, IntAct (Del Toro *et al.* 2022), and BioGRID (Oughtred *et al.* 2021) databases. The network in the Knowledge Graph canvas was constructed based on PPI data.

**Differential expression:** Differential expression results for disease vs. control were calculated based on transcriptomic and Proteomic data. These results are provided in the knowledge base as log2-fold change.

**Target disease association:** numeric scores linking a gene or protein to a given disease are imported from the Open Targets Platform (Ochoa *et al.* 2023). These scoring metrics are integrated into the knowledge base to support downstream analyses of gene-disease associations.

**Target prioritization factors:** numeric metrics imported from the Open Targets Platform are available to help identify promising targets for drug development by considering both the strength of evidence and the likelihood of successful therapeutics.

**Pathway data:** The knowledge base contains complete pathway data from KEGG and Reactome databases. This pathway information can be mapped on the network or executed in real-time gene set enrichment analysis from the interface.

**Druggability data:** Druggability scores are derived directly from DrugnomeAI (Raies *et al.* 2022), which estimates the Druggability likelihood for every protein-coding gene in the human exome.

**Tissue expression data:** Tissue-specific expression calculated from bulk RNA-seq, and cell type-specific expression from single nuclei RNA-seq (Hodge *et al.* 2019) is available in the knowledge base to extract information specific to a given tissue or cell type.

### 2.4 Network visualization and analysis

The core analytic framework of TBEP centers on a knowledge graph constructed from curated biological databases. Using data from PPI networks such as those found in STRING and pathways data from Reactome, TBEP represents essential molecular interactions in a visually intuitive format. To facilitate functional analysis, the platform incorporates gene set enrichment analysis (Subramanian *et al.* 2005, Chen *et al.* 2013, Kuleshov *et al.* 2016) using an upper cutoff value of 0.05 for the adjusted *P*-value, applied against a comprehensive pathway database comprising Reactome and KEGG databases. This approach enables the identification of biological pathways significantly enriched within selected genes, providing insights into underlying molecular mechanisms and functional relevance.

For network community detection, TBEP employs the Leiden algorithm (Newman 2006), which groups nodes into communities based on network topology. Community identification is visually reinforced through a color assignment technique that leverages the golden section within the HSL color model. This approach programmatically produces a palette of deterministic, human-distinguishable colors, ensuring that clusters are easily distinguishable, which improves the clarity of data interpretation.

### 2.5 TBEP assistant—fine-tuned LLM model for biomedical research

Standard LLMs lack the precision, structured output, and verifiable citations needed for specialized biomedical research, often providing generalized, uncited answers. To address this, we developed the TBEP Assistant, a specialized Question Answering (QA) system within TBEP based on a fine-tuned language model.

**Figure 2.**
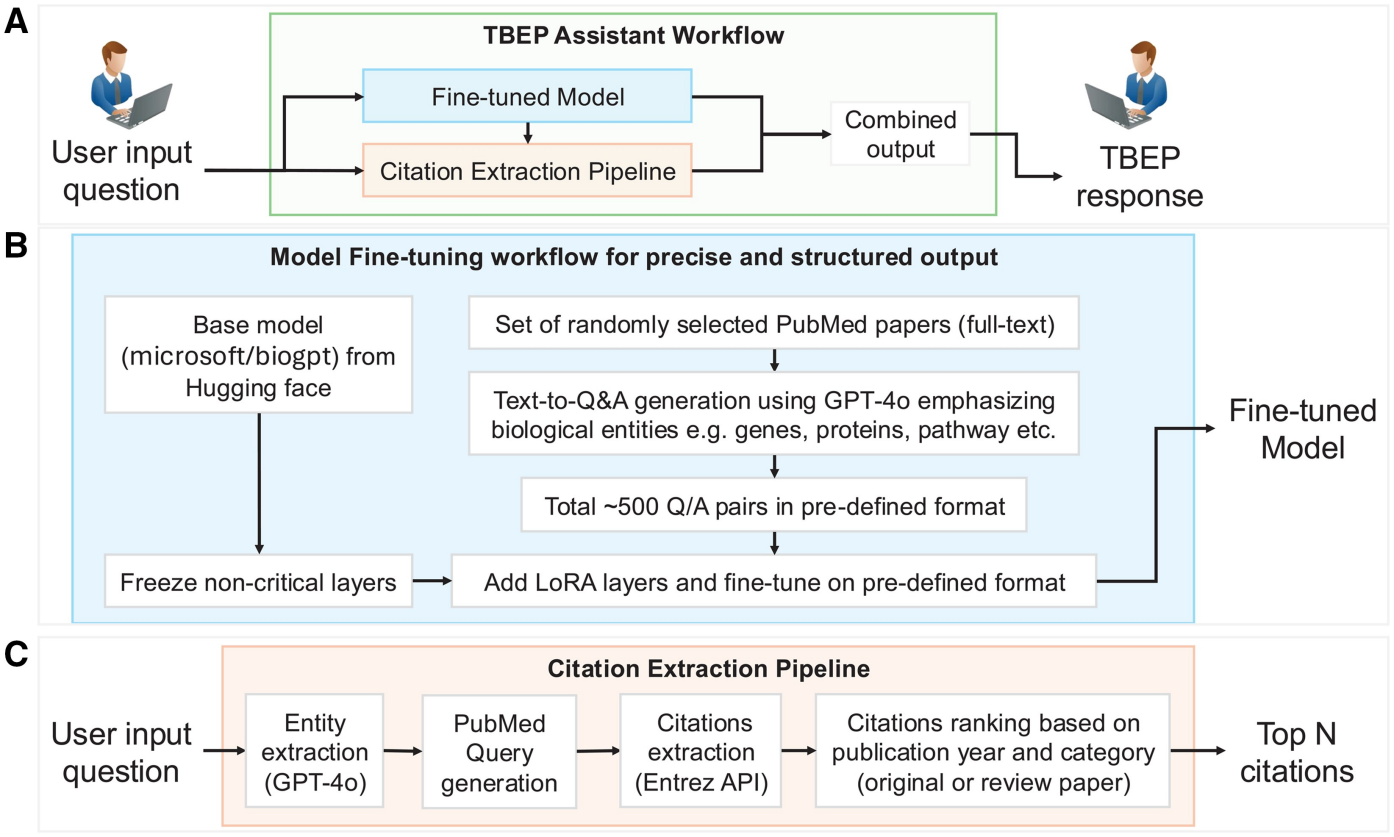
Dual-pipeline architecture of the TBEP Assistant for generating context-aware, reference-supported biomedical responses. (A) Schematic overview of the TBEP Assistant, which integrates two core components: (i) a fine-tuned LLM optimized for generating structured biomedical answers, and (ii) a citation extraction module for retrieving supporting evidence. The outputs of both components are merged to produce accurate and citation-backed responses. (B) Fine-tuning workflow of the BioGPT-based model, incorporating generation of ∼500 question-answer pairs centered on biological entities (e.g. genes, proteins, and pathways), followed by model adaptation using Low-Rank Adaptation (LoRA) techniques to enhance domain-specific response precision. (C) Citation Extraction Pipeline, illustrating key steps including biomedical entity recognition using GPT-4o, automated PubMed query formulation, citation retrieval through the Entrez API, and ranking based on publication type and recency to prioritize the most relevant references.

Specifically, starting with a base model adept in biomedical language (Microsoft/BioGPT from Hugging Face, configurable) (Luo *et al.* 2022), we employed Parameter-Efficient Fine-Tuning (PEFT) (Houlsby *et al.* 2019) using Low-Rank Adaptation (LoRA) (Edward *et al.* 2021) as depicted in Fig. 2B. This involved freezing the non-critical layers of the base model to preserve its extensive pre-trained knowledge while adding lightweight, trainable LoRA layers. The model was then fine-tuned using a novel, manually curated dataset of ∼500 question-answer pairs derived from ∼30 diverse, full-text PubMed publications. Curation focused on queries pertinent to drug discovery and target validation, emphasizing structured entity lists (e.g. genes, proteins, pathway memberships, functional roles, and other corresponding biomedical questions) as answers, thereby ensuring the training data directly reflects the information needs and desired output format for biomedical researchers.

Recognizing the centrality of evidence, the model incorporates an independent Citation Retrieval Module (Fig. 2C). This module uses entity extraction on the user query to formulate optimized searches against the NCBI PubMed database via the Entrez API for live data fetching. Retrieved citations are ranked using a configurable algorithm prioritizing publication type (e.g. reviews or original research) and recency. Development employed iterative refinement, evaluating performance with quantitative metrics on a small subset of validation data, confirming significant gains in structured format adherence over the base model. Qualitative evaluations, including user feedback and structured A/B testing on representative queries, informed successive fine-tuning rounds, ensuring technical robustness and alignment with researcher usability requirements.

Within TBEP, a backend orchestrator manages the QA workflow: directing queries to the fine-tuned model, concurrently initiating citation retrieval and ranking, and integrating the structured textual answer with top-ranked, formatted citations (with PMID/DOI links). This delivers concise, actionable information substantiated by verifiable, current scientific evidence directly within the user interface.

## 3 Results

To develop a systematic framework for *in silico* target and biomarker identification in drug discovery, we implemented a modular analysis workflow within the TBEP. This platform facilitates the integrative analysis of multi-omics datasets, aiming to mitigate study-specific and omics-specific biases in candidate prioritization.

**Figure 3.**
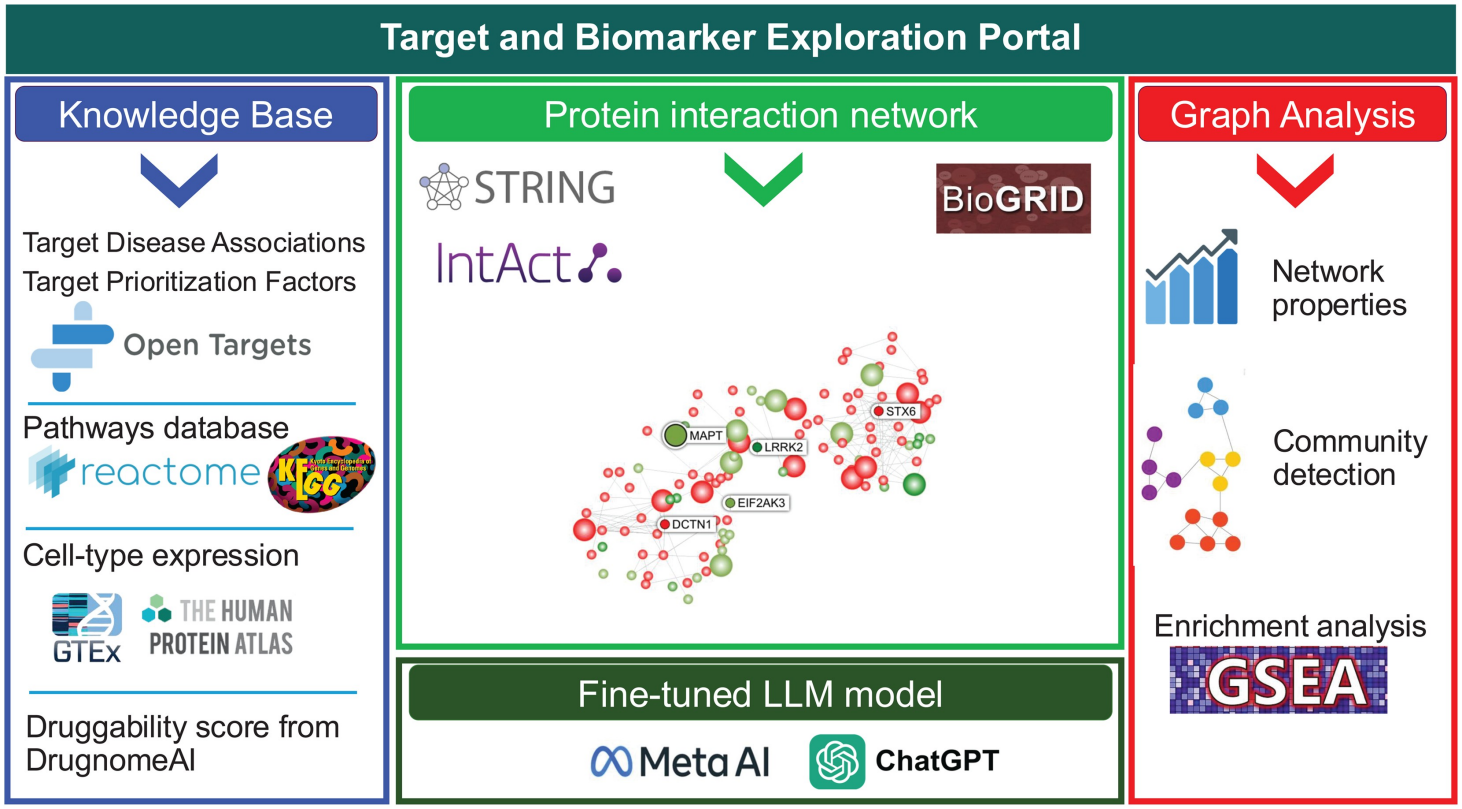
A comprehensive overview of the TBEP architecture. Illustrating an integrative pipeline that combines biological databases, protein interaction networks, and computational analysis. Knowledge Base (left panel): The portal leverages multiple curated biological data sources including OTP, Reactome, KEGG, GTEx, and The Human Protein Atlas to extract relevant gene-disease associations, pathway information, gene expression data, and functional annotations. The tool imports druggability predictions from the published ML-based model DrugnomeAI. Protein Interaction Network (center panel): Using the STRING database, the portal constructs a PPI network centered on selected targets, visualizing molecular interactions to uncover potential functional modules or hubs related to disease mechanisms. Graph Analysis (right panel): Advanced analytical methods are applied to the interaction network, including network topology assessment, community detection, and gene set enrichment analysis (GSEA) to prioritize candidate genes and pathways. Fine-tuned LLM model (bottom panel): A fine-tuned LLM, incorporating tools from Meta AI and ChatGPT, is used to integrate, interpret, and contextualize findings across modules, enhancing the interpretability and translational potential of the portal’s outputs.

As depicted in Fig. 3, the architecture comprises three interrelated components: (i) a curated knowledge base constructed from multiple established bioinformatics repositories, (ii) a protein-centric knowledge graph encoding molecular interaction networks, and (iii) a knowledge bot—an LLM-powered question-answering system designed to support hypothesis generation and evidence synthesis. Collectively, these components enable a data-driven, context-aware strategy for target and biomarker discovery.

### 3.1 Knowledge base—bioinformatics database for query and graph-based analysis

The TBEP knowledgebase is built upon the Neo4j graph database architecture, enabling advanced query and graph-based exploration of bioinformatics data. This database integrates a diverse array of disease-independent datasets. Utilizing the latest versions available from established resources, e.g. GTEx (GTEx Consortium 2013), Human Protein Atlas (Uhlén *et al.* 2015), Brain cell types (Hodge *et al.* 2019), KEGG (Kanehisa and Goto 2000), Reactome pathways (Milacic *et al.* 2024), MSigDB (Subramanian *et al.* 2005), GWAS Catalog (Sollis *et al.* 2023), OpenTargets (Ochoa *et al.* 2023), and Druggability from DrugnomeAI (Raies *et al.* 2022), as well as network data from STRING, and ConsensusPathDB (Herwig *et al.* 2016). Here, disease-independent data refers to the foundational datasets that are not specific to any single disease but provide a broader biological context. For disease-specific data, differential expression data from AMP-AD knowledge portal (Hodes and Buckholtz 2016) is used, which includes differential expression metrics calculated using brain-specific bulk-RNA-seq after preprocessing and harmonizing NGS data from three different brain banks, i.e. MSBB (Wang *et al.* 2018), MayoRNASeq (Allen *et al.* 2016), and ROSMAP (Pérez-González *et al.* 2024). Here, disease-dependent data refers to datasets directly associated with specific diseases or conditions.

### 3.2 Protein interaction network—construction, visualization, and analysis

This module constructs the protein interaction network and maps relevant bioinformatics data for visualization. The module utilizes node color and size attributes to map various bioinformatics disease-dependent and disease-independent data. It also labels and colors the interaction confidence score between any gene pair. The module also provides useful tools for network analysis for candidate selection. For instance, the tool facilitates real-time, interactive gene set enrichment analysis using the Reactome and KEGG pathway databases; employs radial analysis to remove edges based on predefined cutoffs, thereby reducing network complexity and enhancing confidence in candidate identification; and applies clustering algorithms to uncover biologically relevant modules.

### 3.3 TBEP assistant—LLM-based specialized question answering (QA)

**Figure 4.**
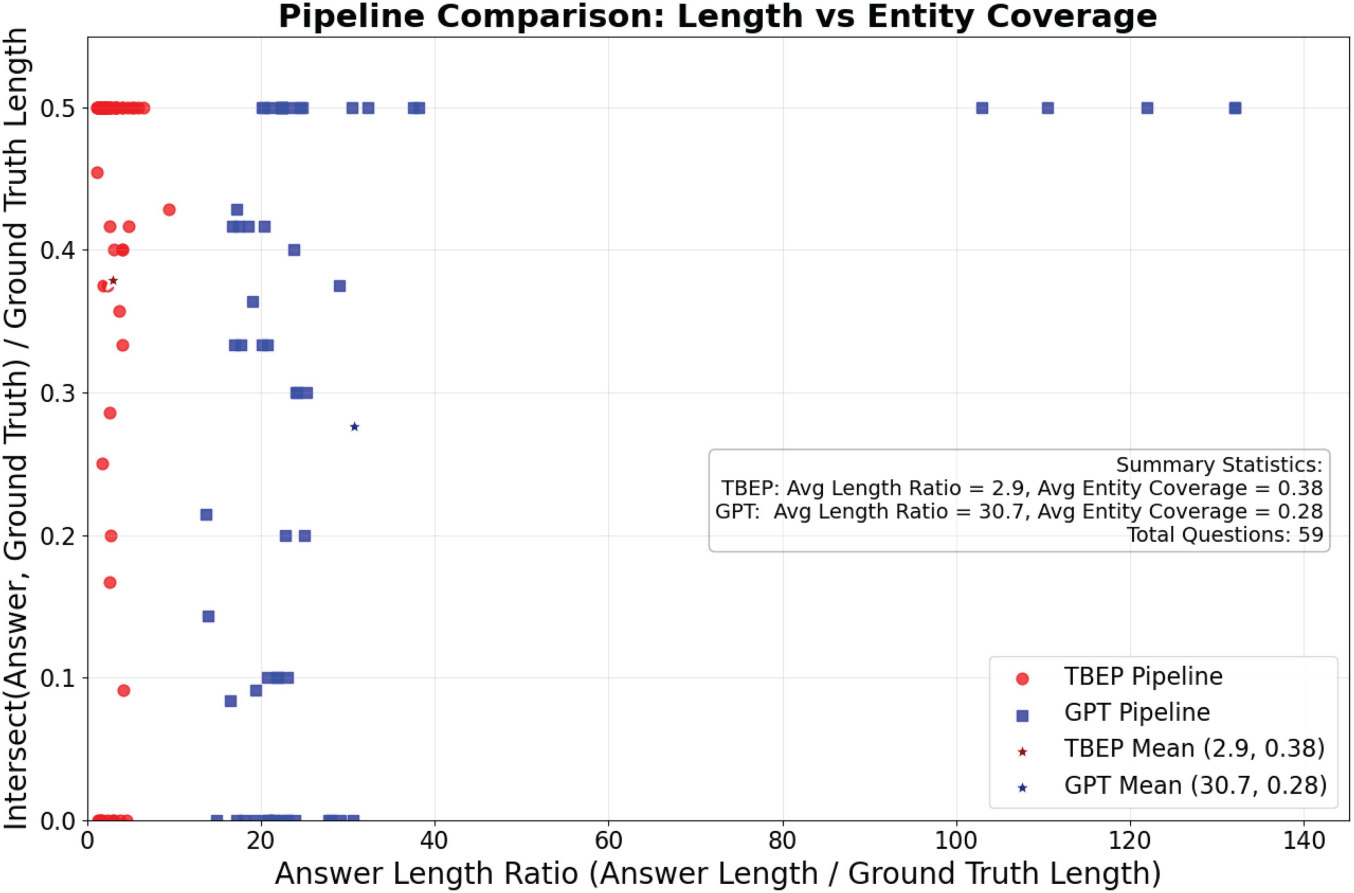
Comparative evaluation of TBEP Assistant and GPT-4.1. The figure shows that TBEP produces more concise, entity-focused, and factually precise responses, aligning with the information density required for biomedical research.

The tool delivers highly factual, concise, and precise answers to specific biomedical questions, outperforming traditional LLMs in terms of precision (Fig. 4; Fig. 1, available as supplementary data at *Bioinformatics* online) because they are trained on the massive English language corpus. For instance, when asked about genes involved in Parkinson’s disease, it directly lists the gene names, disease pathways, and interactions, avoiding generic or vague responses common in traditional models.

It excels at handling questions requiring scientific precision, providing correct and contextually accurate answers, tailored for researchers and scientists. Additionally, the tool grounds its answers with its feature of validating the answers with their corresponding citations, sourced from credible medical databases or web scraping. While citation validation is still under testing, future updates will enable the tool to dynamically recognize and include novel sources. However, the focus remains on delivering accurate, fact-driven responses for scientific use.

To further highlight the model’s precision and information efficiency, Fig. 4 presents a comparative visualization of the answer lengths and factual accuracy produced by the TBEP Assistant versus GPT-4.1. As shown, the fine-tuned model consistently generates shorter, more entity-specific, and factually precise responses—demonstrating its superior suitability for high-accuracy biomedical question answering, where conciseness and verifiable information are critical.

### 3.4 Bioinformatics analysis workflow using TBEP

**Figure 5.**
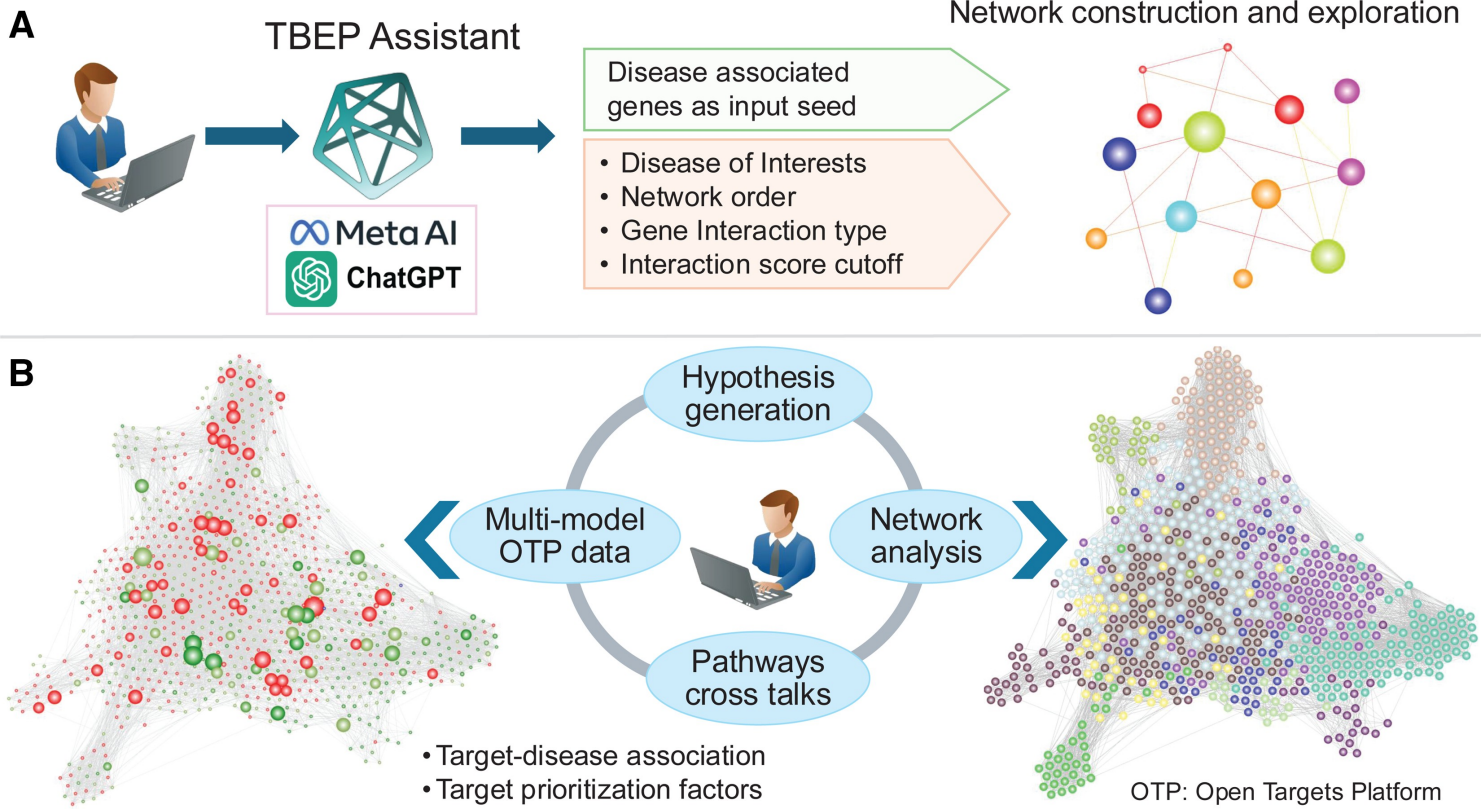
Conceptual workflow for discovering genetically anchored novel therapeutic targets and biomarkers using an LLM-guided network-based approach. This figure outlines a streamlined framework integrating a fine-tuned LLM with multi-omics data to facilitate hypothesis generation in disease research. (A) The user initiates the process by inputting disease-associated seed genes along with customizable parameters, including the disease of interest, network depth, gene interaction types, and interaction confidence thresholds. Users can leverage fine-tuned LLM Assistant to find these inputs to construct a disease-specific gene interaction network tailored for exploration. (B) The generated network is then integrated with diverse omics datasets, such as those from the Open Targets, enabling rich contextualization of gene interactions. Users can interactively explore the network to uncover pathway crosstalk, perform systems-level network analyses, and iteratively generate and refine hypotheses for novel targets and biomarkers grounded in genetic evidence.

To decode causal disease mechanisms for novel therapeutic targets and precision biomarkers, we hypothesized that a genetic mutation causing disease phenotype triggers a cascading chain of biological processes leading to disease-relevant phenotypes by functionally interacting with other genes, which in turn cascade to downstream pathway malfunction. Following our hypothesis, we created a workflow (Fig. 5) to help users explore targets using this hypothesis. Researchers can use fine-tuned LLM agents to find seed genes for a given disease that could be input into the portal along with analysis options. The portal will construct a full-scale protein interaction network, which could be considered as a baseline network as other disease-specific, e.g. differential expression, genetics association, target score, etc. and disease-independent bioinformatics data, e.g. tissue specificity, pathways, etc. could be mapped on this baseline network. The portal provides useful options like disease-relevant module detection and gene set enrichment analysis to select targets and biomarkers.

### 3.5 Quantitative and functional benchmarking of TBEP

**Figure 6.**
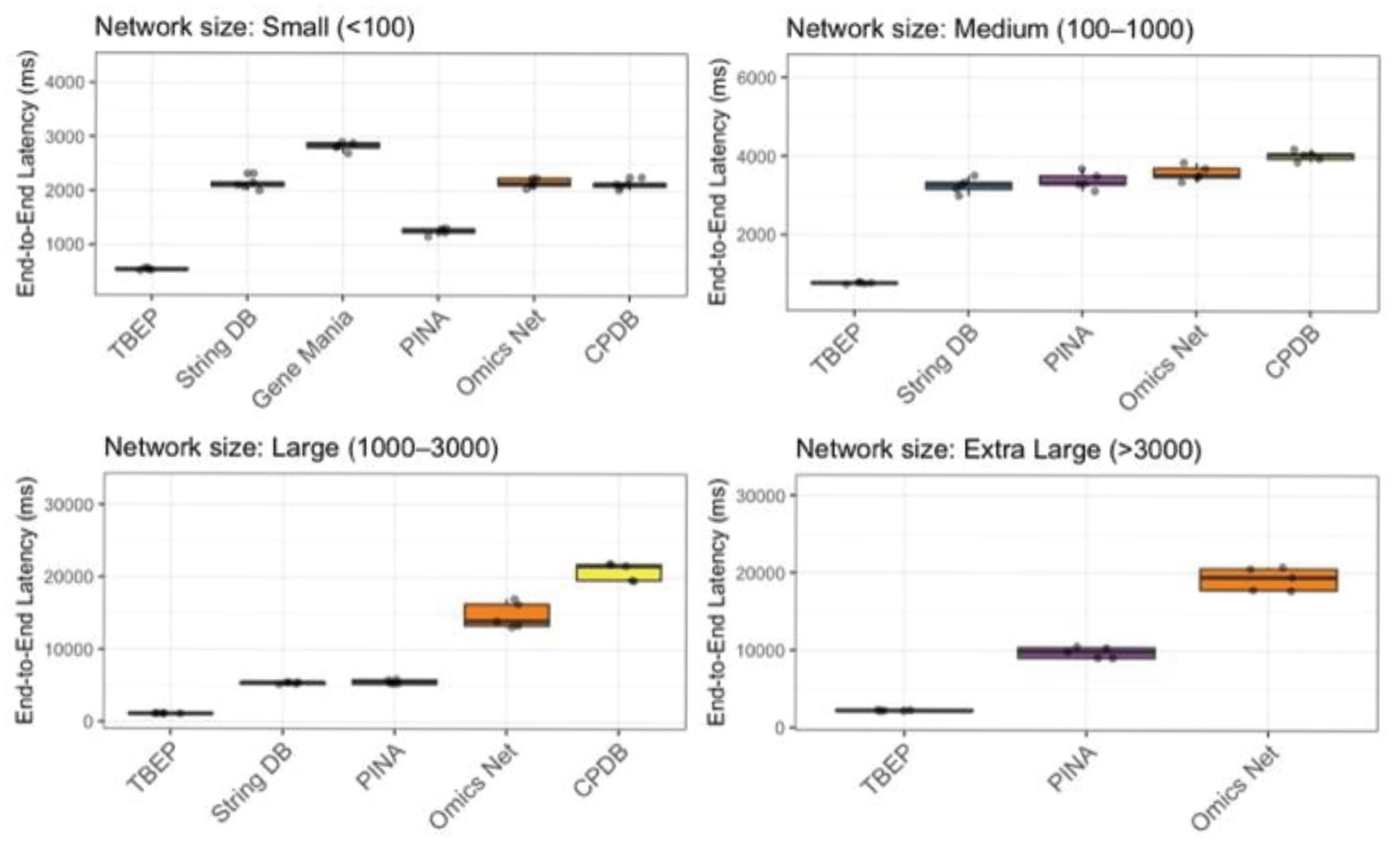
Comparative performance evaluation of TBEP and other existing network analysis tools. The box plot illustrates the response time of various network analysis tools across different network sizes where it shows data of multiple replications. TBEP consistently shows the lowest median latency, indicating superior performance and scalability. While the latency of all the tools increases with network sizes, TBEP’s increase is the most gradual. Only TBEP, PINA and OmicsNet are shown to be scalable to extra-large networks (>3000 nodes), with other tools having limitations in handling larger networks. To know further details on the data used for the plots, refer to the Supplementary Material, available as supplementary data at *Bioinformatics* online.

To substantiate the practical value of TBEP (https://tbep.missouri.edu/) in network biology, we performed a comprehensive benchmarking analysis against several widely used platforms, including STRING (https://string-db.org/), Open Targets (https://platform.opentargets.org/) (Ochoa *et al.* 2023), ConsensusPathDB (http://cpdb.molgen.mpg.de/) (Herwig *et al.* 2016), OmicsNet2.0 (https://www.omicsnet.ca/) (Ewald, *et al.* 2024), GeneMANIA (https://genemania.org/) (Warde-Farley *et al.* 2010), and PINA (https://omics.bjcancer.org/pina/) (Du *et al.* 2021). Specifically, we assessed the response time required to render interaction networks of similar scale across all platforms. To ensure a fair comparison, networks were rendered to fall within defined size categories: Small (<100 nodes), Medium (100–1000 nodes), Large (1000–3000 nodes), and Extra Large (>3000 nodes). As shown in Fig. 6, TBEP consistently outperformed the other tools in response time.

These findings reinforce TBEP’s efficiency and responsiveness while also highlighting its unique strengths against similar tools—including intuitive visualization, integrated gene set enrichment, multi-omics integration, etc. (Table 1, available as supplementary data at *Bioinformatics* online).

### 3.6 Use case: genetically anchored novel targets discovery for Alzheimer’s disease

**Figure 7.**
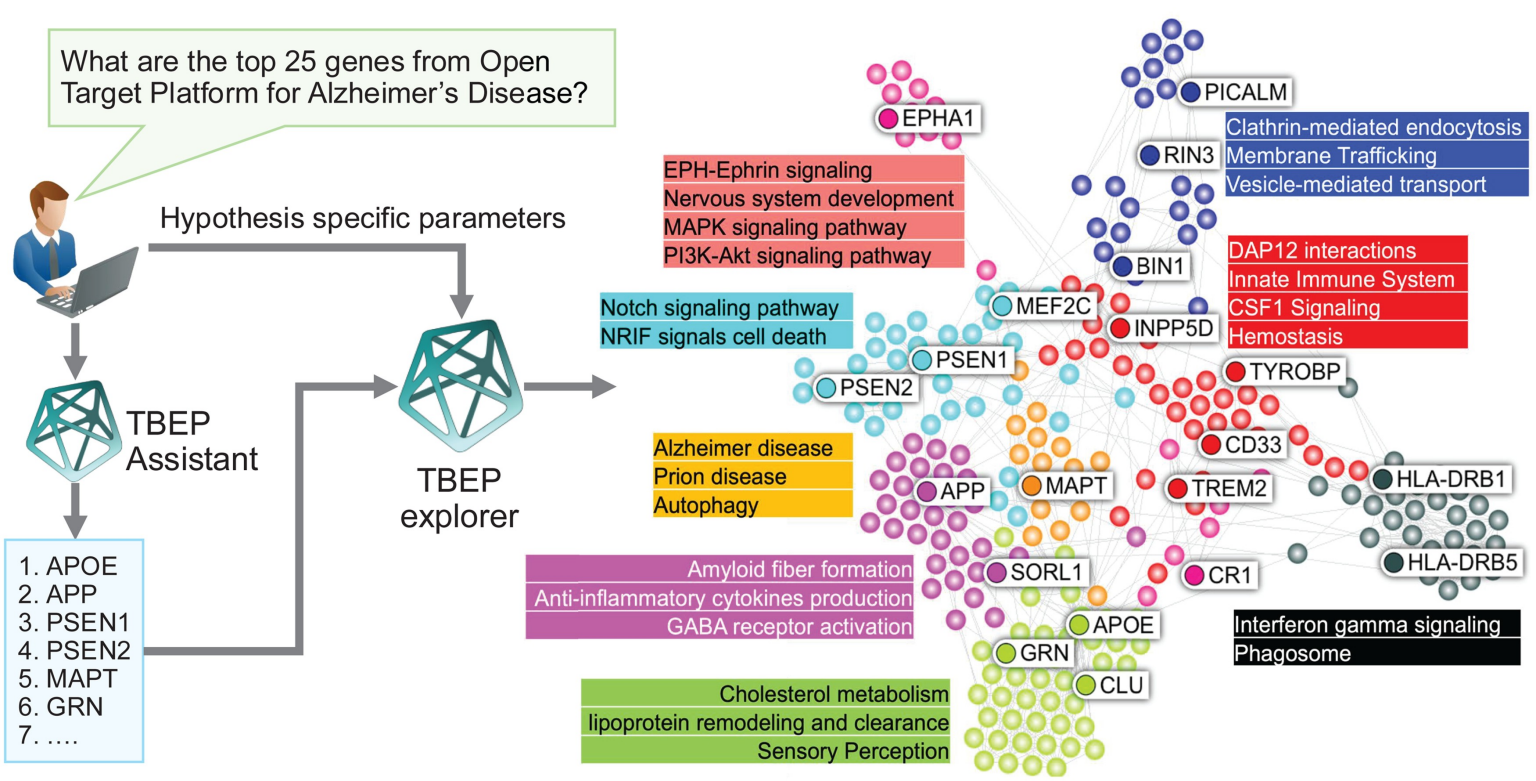
Use-case illustration for deciphering Alzheimer’s disease biology through genetics-anchored pathway mapping and network analysis. The figure demonstrates the utility of TBEP Assistant to extract the top 25 Alzheimer’s disease-associated genes from the Open Targets Platform (OTP), which serve as input seed genes for constructing a disease-relevant protein interaction network. The network visualization reveals distinct functional modules and biological pathways, highlighting pathway crosstalk that underpins disease complexity. Key pathways represented include amyloid fiber formation, immune signaling, vesicle-mediated transport, cholesterol metabolism, and neuronal signaling cascades such as Notch and MAPK. Genes such as APOE, APP, PSEN1, and MAPT anchor critical modules, facilitating the identification of key drivers and interactions for downstream hypothesis generation and target prioritization. This approach enables systematic exploration of genotype-to-phenotype relationships, aiding in the discovery of mechanistically relevant therapeutic candidates.

To showcase the application of TBEP for genetically informed target discovery, we applied it to Alzheimer’s disease (AD) using the prompt: “What are the top 25 genes from the Open Targets Platform for Alzheimer’s Disease?” The retrieved seed genes (Supplementary Data 1, available as supplementary data at *Bioinformatics* online) were used to construct a second-order PPI network via the STRING database, using a high-confidence interaction score threshold (>0.9). The resulting network comprised 246 genes and 776 high-confidence interactions (Fig. 7).

Unsupervised clustering using the Leiden algorithm revealed eight discrete gene communities, each significantly enriched for AD-relevant biological processes, including amyloid fiber formation, autophagy, vesicle-mediated transport, and cholesterol metabolism (Fig. 7; Fig. 2B, available as supplementary data at *Bioinformatics* online). Full enrichment results are provided in Supplementary Data 2–11, available as supplementary data at *Bioinformatics* online. Known AD-associated genes localized to biologically coherent communities: APP, MAPT, and PSEN1/2 were linked to canonical amyloid and tau pathologies; APOE, CLU, and SORL1 to lipid metabolism and Aβ clearance; TREM2 and CD33 to microglial activation and immune response; and BIN1, PICALM, EPHA1, and GRN to intracellular trafficking and Proteostasis.

**Figure 8.**
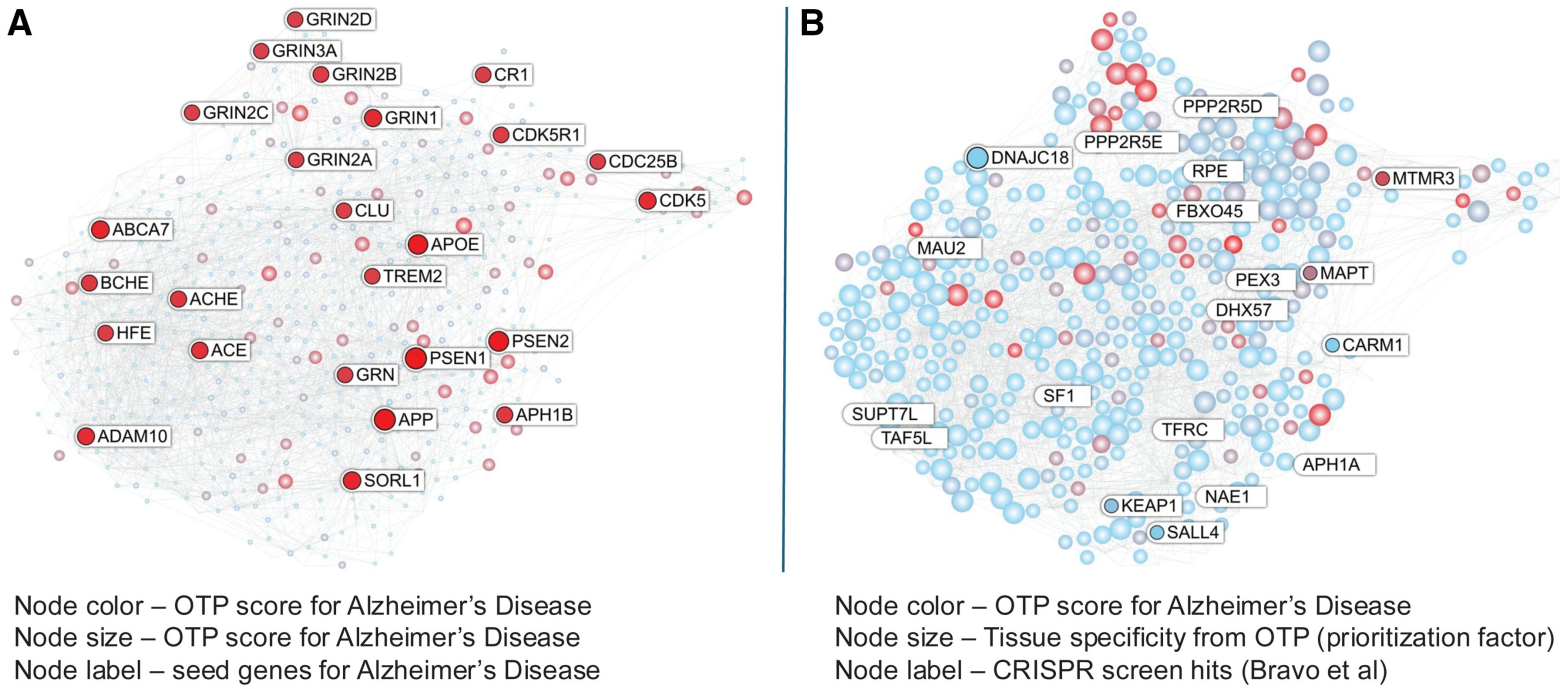
Functional annotation of Alzheimer’s-associated protein interaction network. A protein interaction network was constructed using the top 25 Alzheimer’s disease (AD)-associated genes identified from the Open Targets Platform. (A) Node color reflects tissue specificity, while node size indicates druggability scores, highlighting genes with potential for therapeutic intervention in relevant tissues. (B) Node color indicates whether the gene is a known clinical target, and node size corresponds to the strength of AD genetic association. This integrative network visualization supports prioritization of targets based on multi-dimensional evidence, including tissue relevance, druggability, clinical status, and genetic linkage to AD.

To enable multidimensional prioritization, we overlaid disease-relevant attributes onto the network: node color reflects CRISPR perturbation scores for Alzheimer’s Disease from the Open Targets platform, while node size represents druggability via DrugnomeAI “tclin” scores, i.e. presence as an approved drug target (Fig. 8A; Figs 2C–G, available as supplementary data at *Bioinformatics* online). Genes with both high perturbation scores and large node sizes emerge as high-priority candidates for therapeutic development or repurposing, illustrating the TBEP capacity to guide hypothesis generation for drug repurposing and novel target discovery in AD.

Figure 8B presents a complementary view, where node color represents inclusion in clinical trials (irrespective of indication), and node size highlights AD-specific associations from the Open Targets Platform. Genes that are both AD-associated and in active clinical evaluation stand out as especially promising candidates for further prioritization.

The expanded network was further enriched with functional genomics data from a human iPSC 4R tauopathy CRISPR screen (Parra Bravo *et al.* 2024), which identified genes acting as positive or negative modifiers of tau propagation. These modifier gene lists were overlaid onto the network and highlighted for visual emphasis. Node color represented the Alzheimer’s disease association score from the Open Targets Platform, while node size reflected key target prioritization attributes, including druggability, gene essentiality, and tissue specificity.

Among the highlighted genes, DNAJC18 emerged as a notable candidate due to its high tissue specificity, as indicated by its enlarged node size. In the CRISPR dataset, DNAJC18 was reported as a negative modifier, where gene knockdown suppressed tau seeding, implying a potential role in facilitating tau spread under physiological conditions. Although absent from the Open Targets Alzheimer’s disease gene list, TBEP prioritized DNAJC18 based on its strong tissue specificity and functional relevance in tau pathology.

KEAP1, identified in the CRISPR screen as a positive modifier of tau aggregation, was similarly highlighted by TBEP for its strong gene essentiality and small molecule druggability. As a central regulator of the NRF2-mediated antioxidant response, KEAP1’s emergence underscores TBEP’s ability to integrate network propagation with orthogonal evidence to surface biologically relevant potential novel targets. CARM1 was another high-priority candidate, prioritized for its favorable druggability metrics, including a “Tclin and Tier1” classification in DrugnomeAI, indicating that it is the target of an approved drug. Its presence in the network despite lacking prior AD association suggests potential for drug repurposing.

Collectively, DNAJC18, KEAP1, and CARM1 represent novel targets identified through TBEP’s integration of genetically anchored networks, functional genomics, and target prioritization features as druggability, tissue specificity, and gene essentiality. Their identification in this case study illustrates TBEP’s strength in supporting hypothesis generation for both novel target discovery and drug repurposing in complex neurodegenerative disorders such as Alzheimer’s disease.

While these findings underscore the utility of TBEP in uncovering genetically anchored and druggable targets in AD, further validation is warranted to assess their biological significance. In cases where experimental validation is infeasible, cross-referencing TBEP-identified genes with curated datasets of Alzheimer’s disease (AD)-associated genes may provide additional support for their relevance to neurodegenerative disease biology.

## 4 Discussion

TBEP provides a unique platform for biomedical researchers to explore biological networks with multi-omics data for the systematic identification and prioritization of candidate drug targets and biomarkers. By moving beyond conventional differential expression analyses, TBEP facilitates the discovery of proteins that are functionally central within disease-specific networks, even when such proteins are not differentially expressed at the transcriptomic or protein level. This network-based perspective enables a more comprehensive characterization of disease mechanisms, capturing key regulatory nodes that may otherwise be overlooked.

Critically, the predictions generated by TBEP have considerable translational potential. In preclinical research settings, these predictions can be leveraged to guide the selection of novel therapeutic targets for experimental validation. The integration of TBEP outputs with high-throughput functional genomics platforms, such as CRISPR-Cas9 loss-of-function screens, RNAi assays, or phenotypic compound screens, could enhance early-stage drug discovery by prioritizing targets with strong biological plausibility and network centrality. In the clinical domain, proteins prioritized through TBEP may serve as candidate biomarkers for prognosis, therapeutic response, or disease subtyping. The integration of TBEP analyses with clinical trial datasets, particularly those involving multi-omics profiling, could support the identification of predictive biomarkers or elucidate mechanisms of drug resistance.

Despite its strengths, TBEP is subject to limitations inherent to the underlying data sources. The completeness and reliability of PPI networks remain variable, and the integration of omics datasets from diverse platforms introduces challenges related to data normalization, batch effects, and noise. As such, the accuracy of target prioritization is contingent upon rigorous data preprocessing and the continuous curation of high-confidence interaction networks.

Our ongoing development roadmap includes fine-tuning the underlying models using an expanding corpus of biomedical literature, experimental data, and human-curated annotations. We plan to implement Reinforcement Learning from Human Feedback (RLHF) and domain-specific reward modeling to improve the interpretability and utility of the system for biomedical end-users. These efforts will ensure that TBEP continues to evolve as a translational informatics platform capable of supporting hypothesis generation, target validation, and clinical decision-making across the drug development continuum.

## Supplementary Material

btaf627_Supplementary_Data

## Data Availability

The source code for the tool, along with documentation and installation instructions, is available at the GitHub repository: https://github.com/mizzoudbl/tbep. The web-based version of the tool can be accessed https://tbep.missouri.edu/. Docker image: https://github.com/orgs/mizzoudbl/packages?repo_name=tbep. Zenodo repository DOI https://doi.org/10.5281/zenodo.16742136. All the data curated to build the database and source code are uploaded in this Zenodo release (v1.2.7) https://zenodo.org/records/16746052. The following DOI, 10.5281/zenodo.16742136, redirects to the latest release version of the TBEP tool.

## Supplementary data

Conflict of interest: The authors declare the following potential conflicts of interest: G.S. is a full-time employee of Alexion Pharmaceuticals Inc, AstraZeneca Rare Disease Unit and serves as adjunct faculty in the Department of Electrical Engineering and Computer Science at the University of Missouri-Columbia. This research was conducted as part of an academic collaboration and does not necessarily reflect the views or interests of Alexion, AstraZeneca. Work for the study was done without any use of Alexion’s resources and was conducted exclusively during non-working hours.
