# Supplementary material for: Target and biomarker exploration portal for drug discovery
Source: Bioinformatics. 2025 Nov 13;41(12):btaf627. doi: 10.1093/bioinformatics/btaf627 (PMC12684723; doi:10.1093/bioinformatics/btaf627)
Supplement: btaf627_Supplementary_Data [file btaf627_supplementary_data.docx]

**Supplementary Materials**

**Target and Biomarker Exploration Portal for Drug Discovery**

Bhupesh Dewangan^1^, Debjyoti Ray^1^, Yijie Ren^2^, Shraddha Srivastava^1^, Lei Jiang^2^, Muneendra Ojha^1^, Dong Xu^2*^, Gyan Srivastava^2,3,*^

^1^Indian Institute of Information Technology, Allahabad, India, ^2^Department of Electrical Engineering and Computer Science and Christopher S. Bond Life Sciences Center, University of Missouri, Columbia, Missouri, USA, ^3^Bioinformatics and Data Science, Alexion Pharmaceutical, AstraZeneca, Boston, Massachusetts, USA.

*Corresponding authors:

Dong Xu, [xudong@missouri.edu](mailto:xudong@missouri.edu)

Gyan Srivastava, [gps8b9@missouri.edu](mailto:gps8b9@missouri.edu)

**Supplementary Table 1: Benchmarking TBEP against established network analysis platforms**. The table highlights the unique advantages of TBEP compared to other tools. These features collectively underscore TBEP’s value as a rapid, reliable, and intuitive platform for network-based biological discovery.

|  | **TBEP** | **STRING** | **OTP** | **CPDB** | **ON** | **GM** | **PINA** |
| --- | --- | --- | --- | --- | --- | --- | --- |
| Big network visualization | Yes | No | No | No | Yes | No | Yes |
| Enrichment analysis | Yes | Yes | No | Yes | Yes | Yes | Yes |
| Topology features | Yes | Yes | No | No | Yes | No | Yes |
| Network expansion | Yes | Yes | No | Yes | Yes | Yes | Yes |
| Target selection | Yes | No | Yes | No | No | No | No |
| Druggability inference | Yes | No | Yes | No | No | Yes | No |
| DEG analysis | Yes | No | Yes | No | Yes | Ni | No |
| Modules detection | Yes | Yes | No | Yes | Yes | Yes | Yes |
| Fine-tuned LLM agent | Yes | No | No | No | No | No | No |

**Supplementary Figure 1: TBEP Assistant, Fine-tuned LLM model for scientific research**

The LLM-based knowledge bot delivers highly factual, concise, and precise answers to specific biomedical questions, outperforming traditional large language models in terms of precision and structured responses.

### **
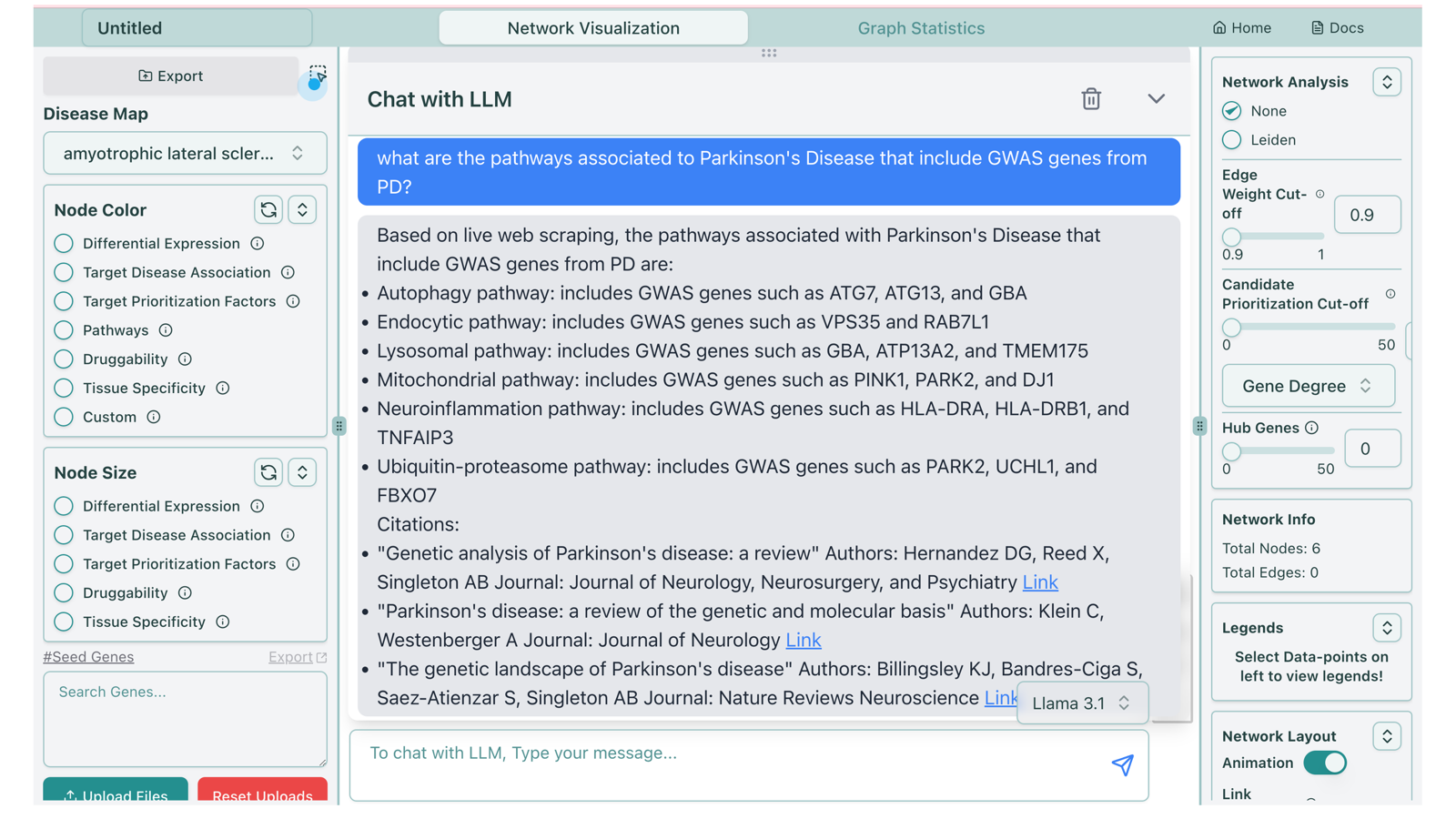
**

### **Supplementary Figure 1A:** Screenshot of the Q/A response from the knowledge bot for the user input question “What are the pathways associated with Parkinson's Disease that include GWAS genes from PD?”

### **
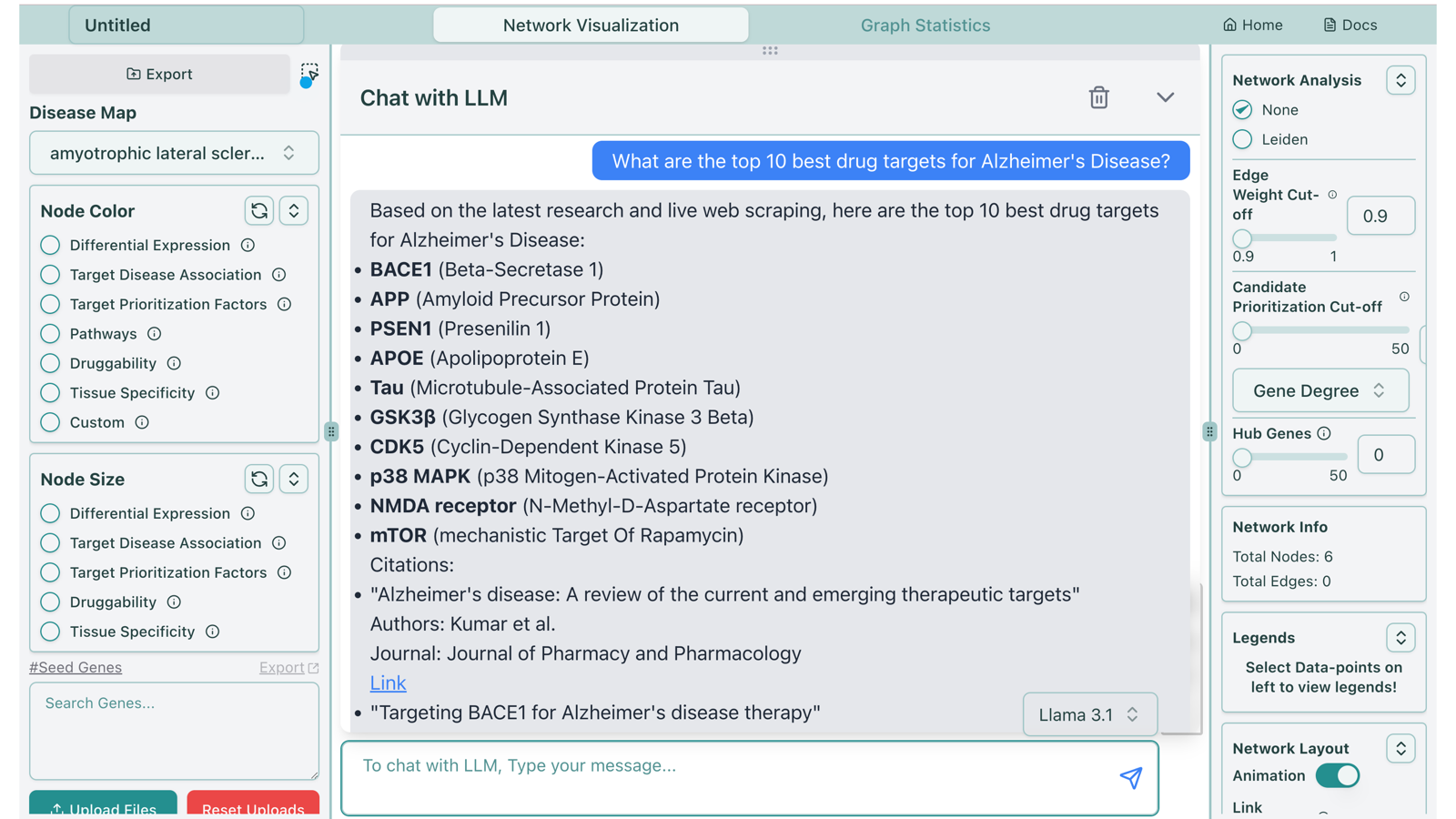
**

### **Supplementary Figure 1B:** Screenshot of the Q/A response from knowledge bot for user input question “What are the top 10 best drug targets for Alzheimer’s Disease?”

### **Supplementary Figure 2: Genetically Anchored Novel Targets for Alzheimer’s Disease**

The TBEP Assistant was employed to identify genetically anchored targets for Alzheimer’s Disease using the "What are the top 25 genes from the Open Target Platform for Alzheimer’s Disease?" prompt. This query generated a list of seed genes, which were then used to build protein-protein interaction (PPI) networks with interaction scores greater than 0.9. A detailed description of these seed genes is provided in Supplementary Data 1.


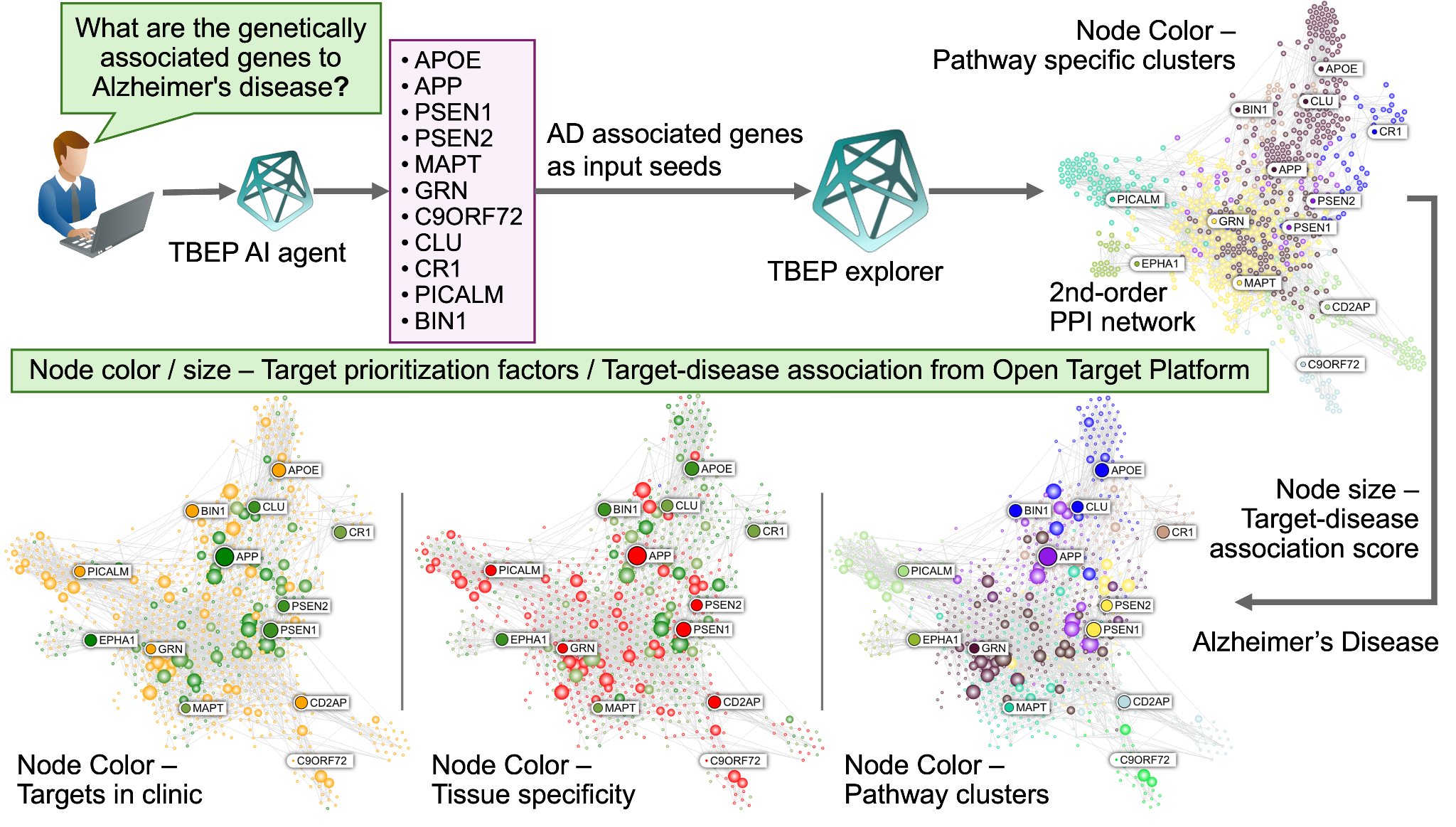


**Supplementary Figure 2A:** Workflow depicting a use case to utilize TBEP to find novel targets and biomarkers for Alzheimer’s disease that are anchored in human genetics.


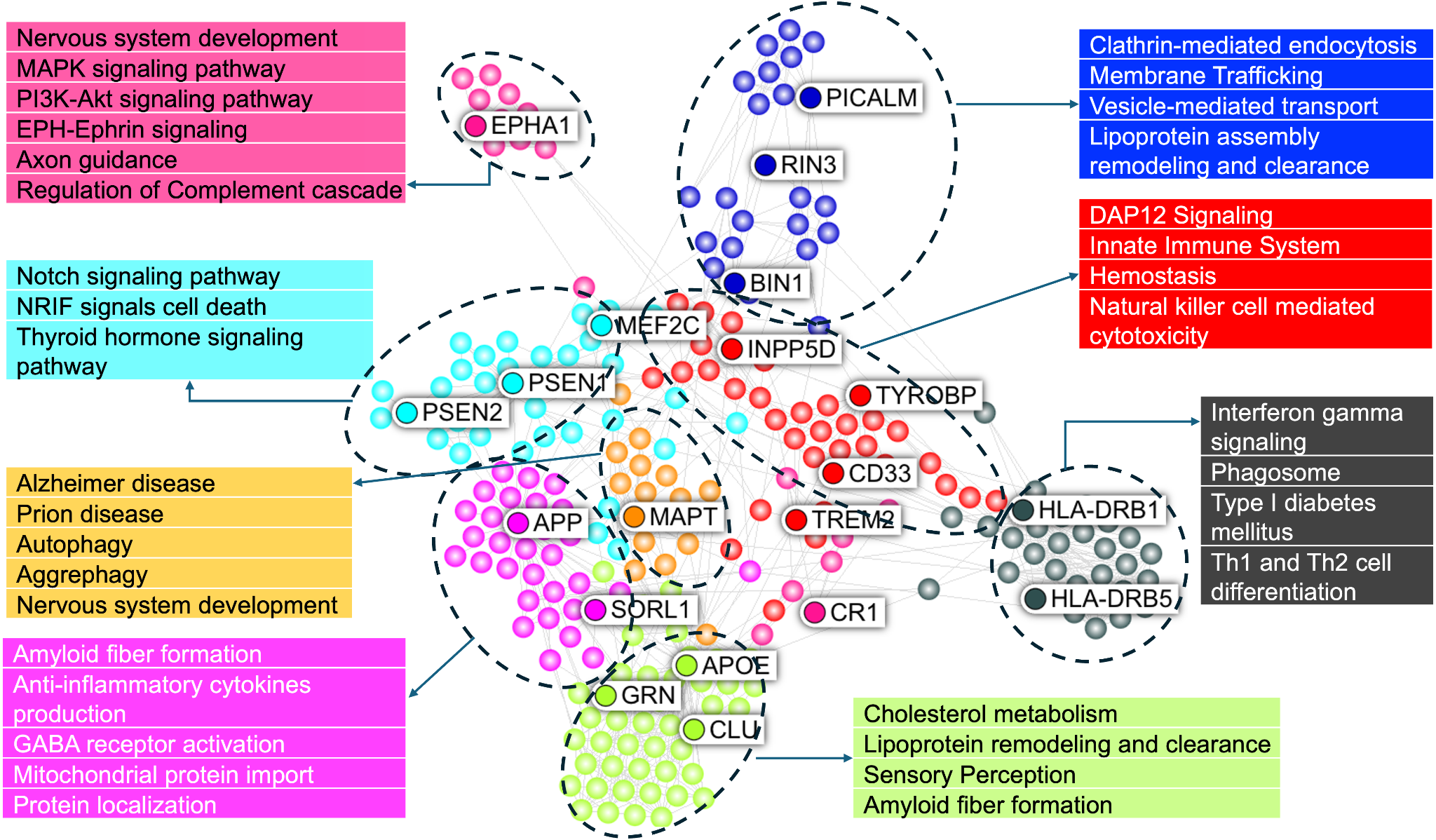


**Supplementary Figure 2B:** Visualization depicting pathway crosstalk among PPI clusters derived from genes interacting with the top 25 seed genes associated with Alzheimer's disease (AD). Unsupervised clustering performed using the Leiden algorithm identified 8 distinct communities within the network. These communities were enriched with biologically relevant pathways, such as amyloid fiber formation and cholesterol metabolism, which are both critical to the pathophysiology of Alzheimer’s disease. More detailed pathway information is available in Supplementary Data 2-11.

### **Key pathway clusters & their relevance to AD**

#### 1. EPHA1 (Pink) Cluster — implicated in synaptic function and axon guidance.

#### 2. PSEN1, PSEN2 (Cyan) Cluster — implicated in Notch signaling disruption and Aβ accumulation. PSEN1/2 are part of γ-secretase, involved in amyloid precursor protein (APP) processing and Aβ generation.

#### 3. MAPT, APP (Orange) Cluster — Encompasses tau (MAPT) and APP, whose dysfunction results in neurofibrillary tangles and amyloid plaques, the two hallmarks of AD.

#### 4. SORL1, GRN (Purple) Cluster — GABA signaling, mitochondrial protein import. Genes like SORL1 affect APP trafficking, influencing Aβ production.

#### 5. BIN1, PICALM, RIN3 (Blue) Cluster — enriched with genes involved in Endocytosis, vesicle transport, and lipoprotein remodeling. These pathways influence Aβ clearance and synaptic vesicle cycling.

#### 6. TREM2, TYROBP, CD33 (Red) Cluster — associated with Innate immunity, DAP12 signaling. Microglial genes (TREM2, CD33) regulate the immune response and phagocytosis of Aβ. TREM2 mutations impair microglial response, exacerbating amyloid pathology.

#### 7. HLA-DRB (Dark Gray) Cluster — represents Interferon signaling, Th1/Th2 differentiation and reflects neuroinflammation and immune modulation, contributing to AD progression.

#### 8. APOE, CLU (Light Green) Cluster — implicated in Cholesterol metabolism, amyloid fiber formation. CLU and APOE modulate Aβ clearance and lipid transport. Notably, APOE ε4 is the strongest genetic risk factor for late-onset AD.


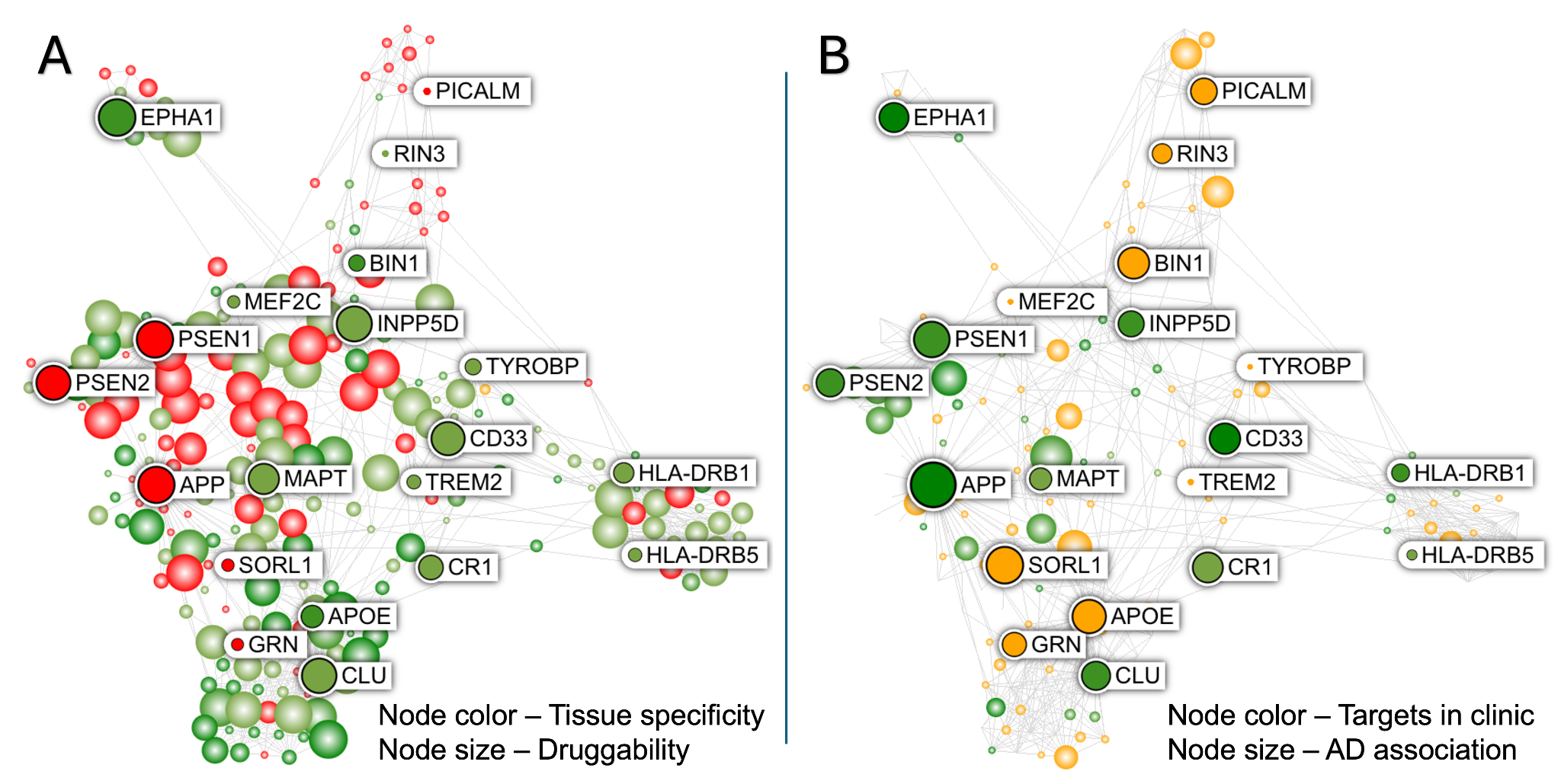


**Supplementary Figure 2C:** Left half network (A) and right half network (B) provide visualizations of the network where node color and size represent different attributes. Left-side network highlights tissue specificity (color) and druggability scores (size), emphasizing genes relevant to specific tissues, while the right-side network uses node color to indicate clinically known drug targets and node size to represent the strength of the gene’s association with Alzheimer’s disease.

**Supplementary Figures 2D–2G** present the network with a focus on disease-dependent attributes, such as differentially expressed genes in disease vs. control samples from the Expression Atlas, as well as GWAS associations. Additionally, these figures incorporate disease-independent attributes, including targets associated with curated adverse events and tissue specificity, which provide further context for target validation.


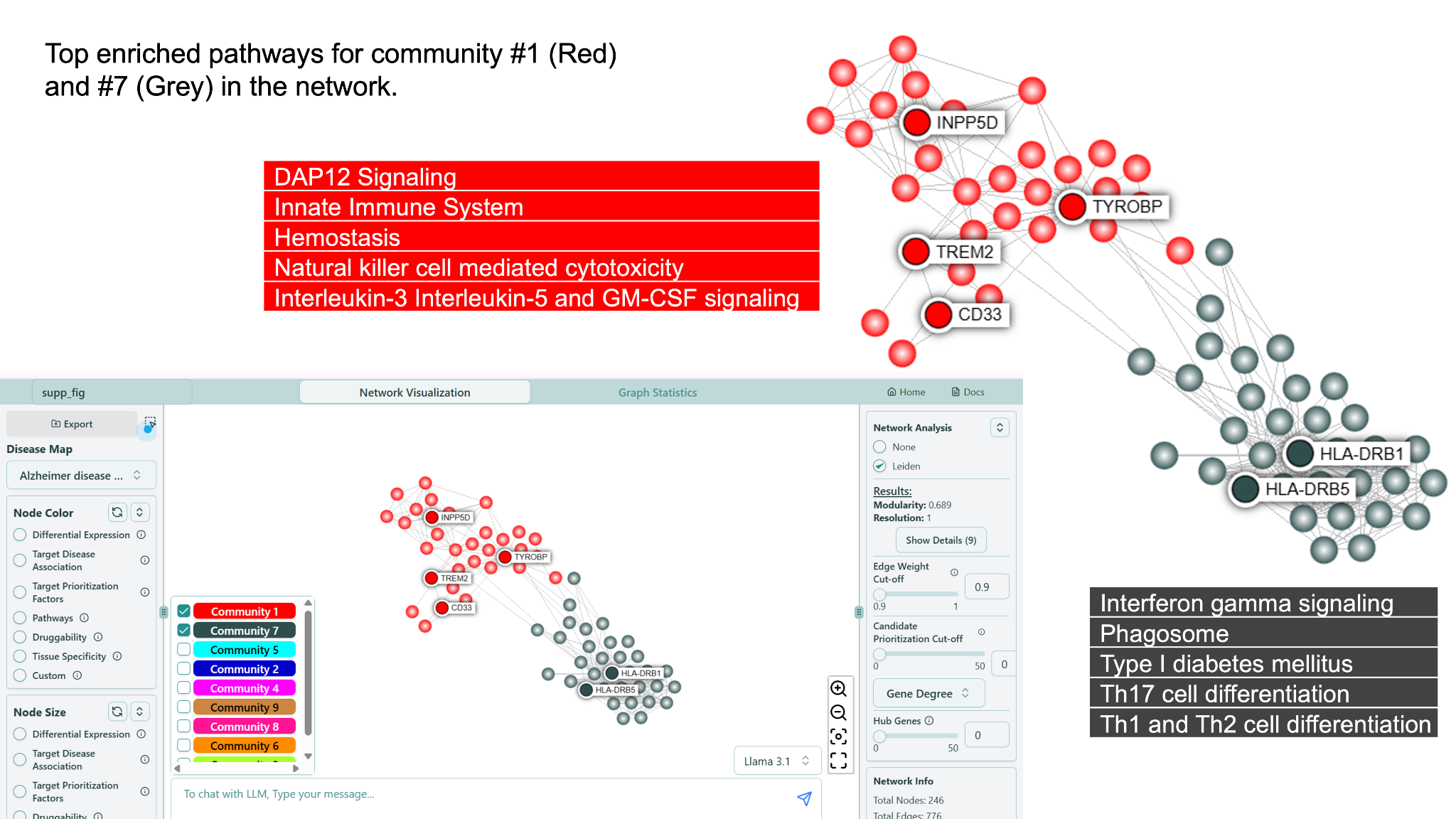


**Supplementary Figure 2D:** top enriched pathways in cluster #1 (Red) and cluster #7 (Grey).


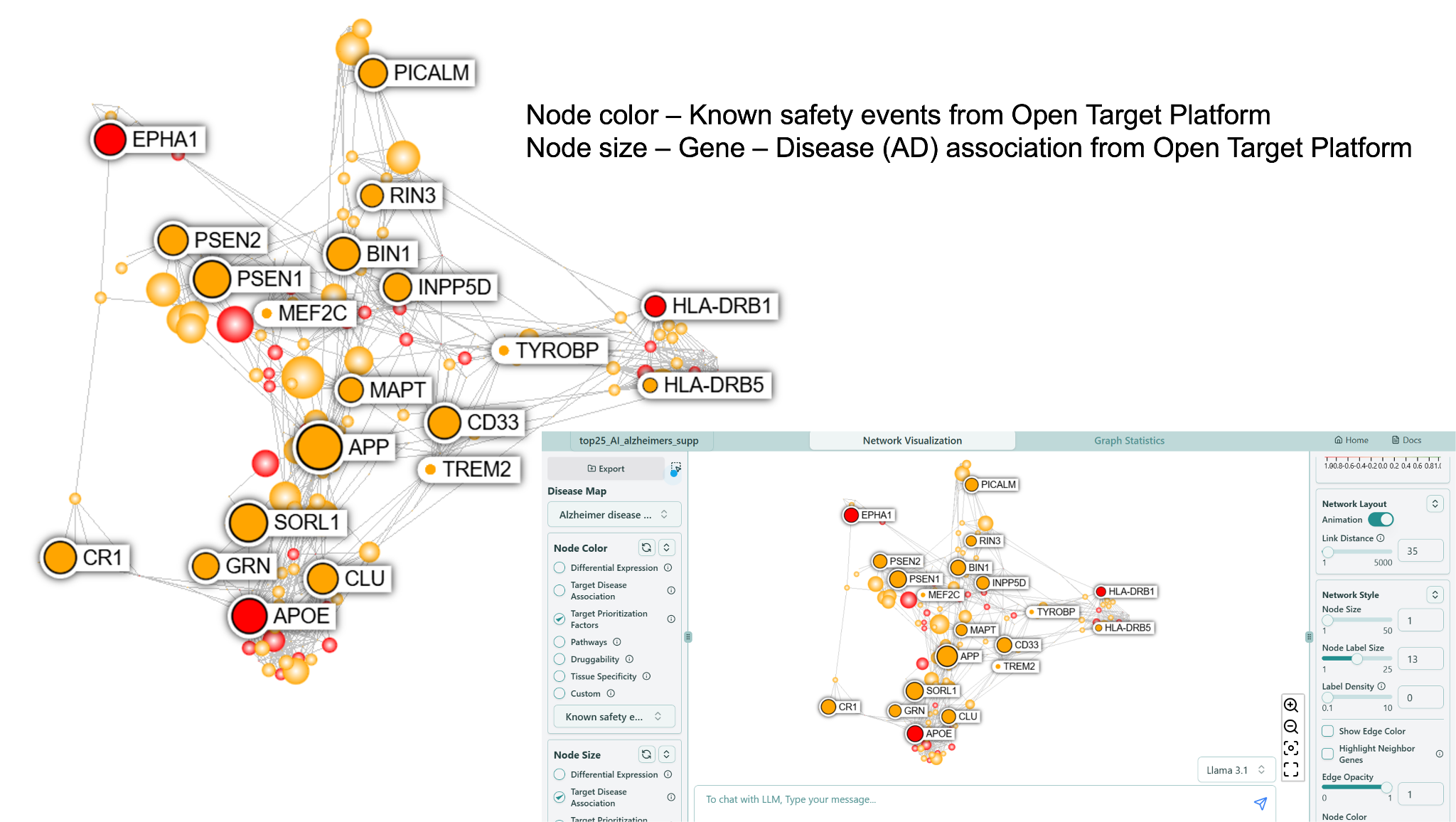


**Supplementary Figure 2E:** Network screenshot mapped with data from the Open Target Platform.


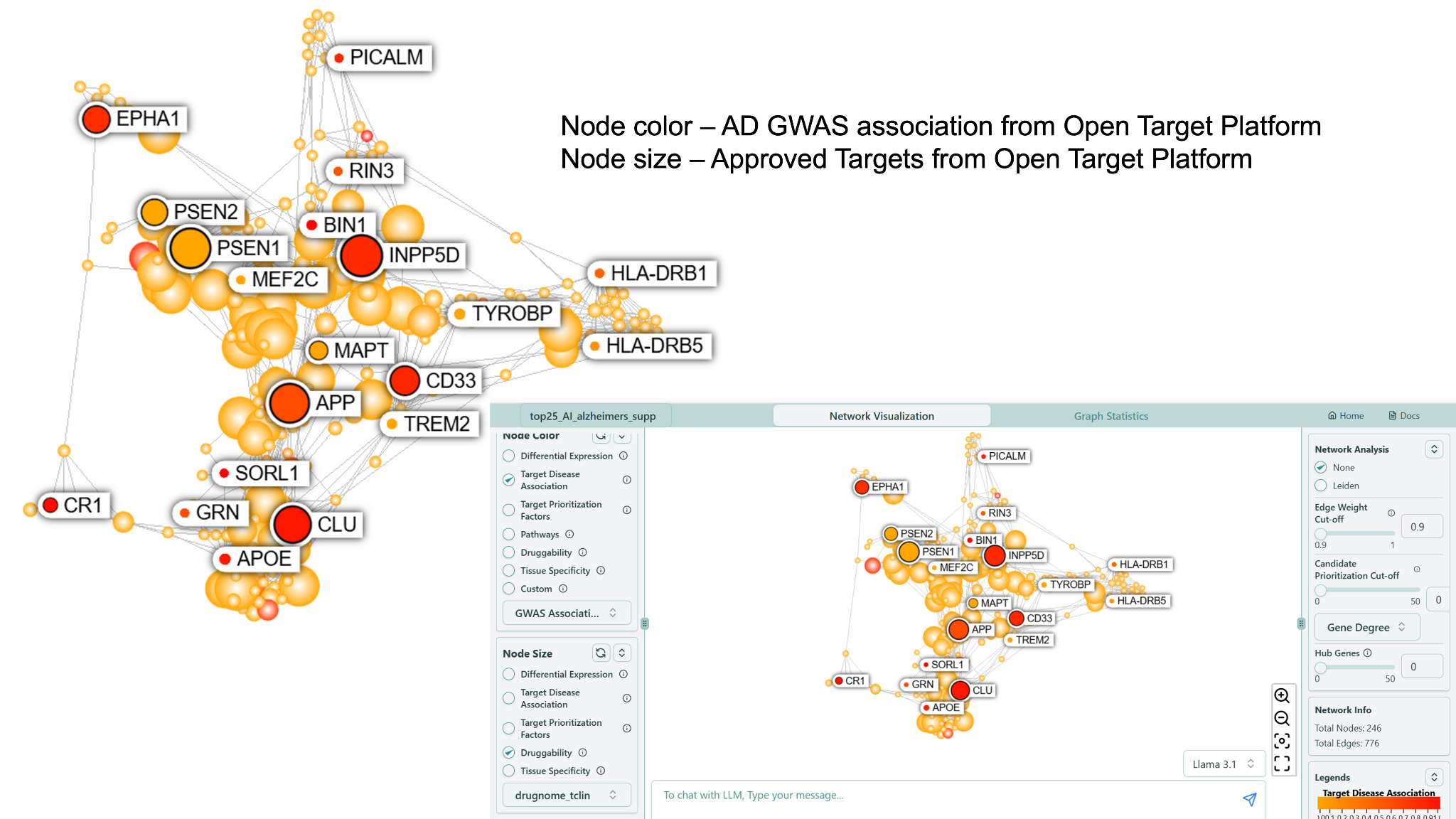


**Supplementary Figure 2F:** Network screenshot mapped with data from the Open Target Platform.


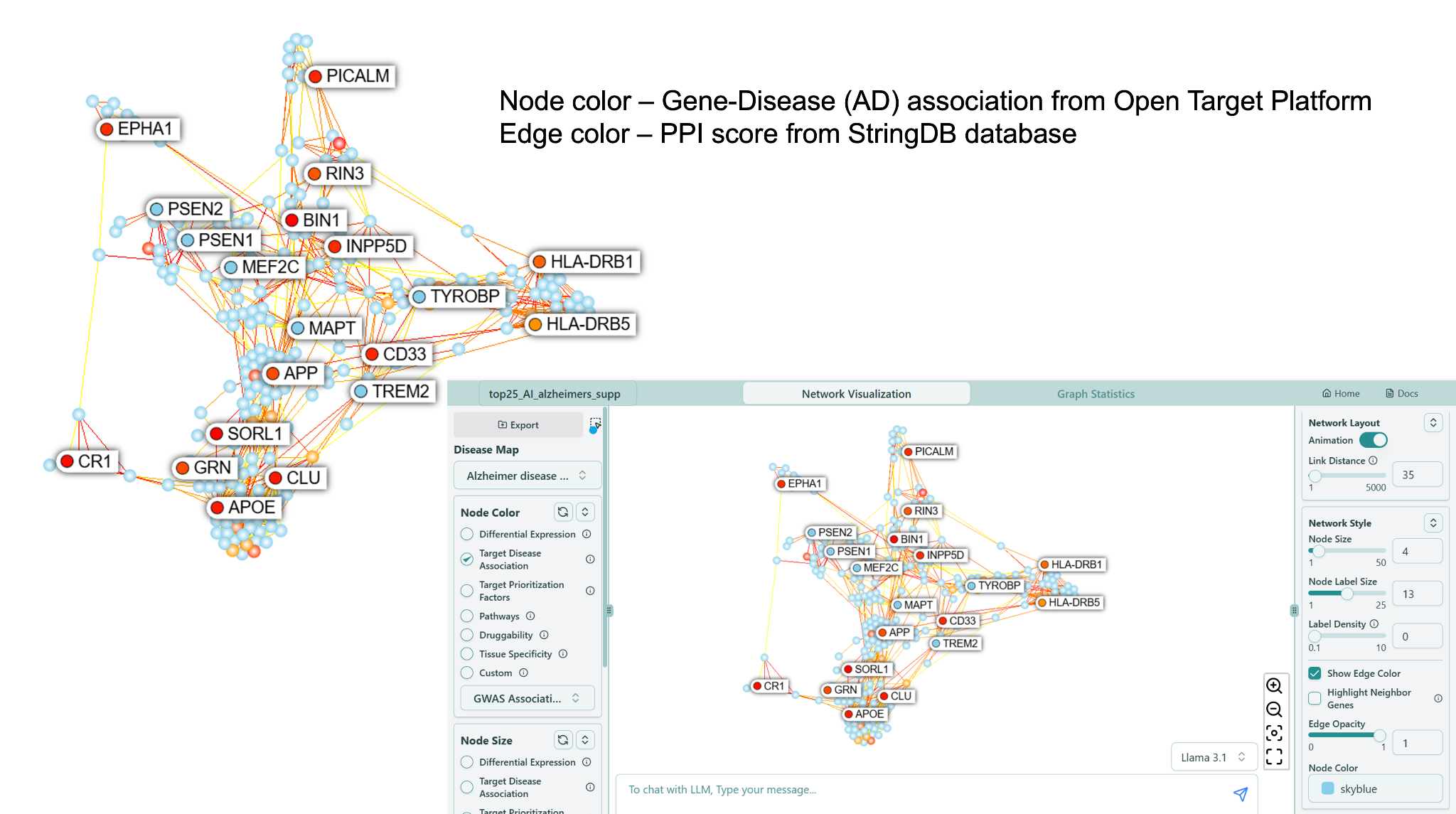


**Supplementary Figure 2G:** Network screenshot mapped with data from the Open Target Platform.
